# Can slow and (un)steady still win the race? A narrative review of developmental gait characteristics and clinical implications in children born preterm

**DOI:** 10.1007/s00431-026-07252-3

**Published:** 2026-07-20

**Authors:** Martina Ruscelli, Anna Fetta, Ettore Benvenuti, Maria Cristina Bisi, Rita Stagni, Elena Zardi, Alessandra Sansavini, Luigi Corvaglia, Duccio Maria Cordelli, Arianna Aceti

**Affiliations:** 1 Department of Medical and Surgical Sciences, University of Bologna, Bologna, Italy; 2 UOC Neuropsichiatria Dell’età Pediatrica, IRCCS Istituto Delle Scienze Neurologiche Di Bologna, Bologna, Italy; 3 Department of Electrical, Electronic, and Information Engineering “Guglielmo Marconi”, University of Bologna, Bologna, Italy; 4 Physical Medicine and Rehabilitation Unit, IRCCS AOU Bologna, Bologna, Italy; 5 Department of Psychology “Renzo Canestrari”, University of Bologna, Bologna, Italy; 6 Neonatal Intensive Care Unit, IRCCS AOU Bologna, Bologna, Italy

**Keywords:** Prematurity, Independent walking, Gait assessment, Inertial measurement units, Neurodevelopmental follow-up

## Abstract

Independent walking is more than a developmental milestone, as it reflects the integration of neuromuscular maturation, postural control, and sensorimotor coordination. In children born preterm, both the timing and the characteristics of independent walking may differ from those observed in term-born peers. This narrative review synthesizes current evidence on independent walking acquisition and gait features in preterm-born children throughout different stages of life, from toddler to school age, and discusses the clinical implications of quantitative gait assessment, with a focus on innovative wearable technologies, during neurodevelopmental follow-up. Preterm birth is consistently associated with a 1–2-month delay in independent walking onset compared with term-born peers, even after correction for gestational age (GA), with greater delays reported in infants born at lower GA or with very low birth weight (BW). Beyond milestone attainment, both qualitative and quantitative studies describe less mature gait patterns during early walking and the preschool years, including shorter step length, wider base of support, prolonged double-support phase, increased temporal variability, and reduced gait complexity. These characteristics likely reflect compensatory strategies related to immature postural control and sensorimotor organization. Although many spatiotemporal parameters improve with age and walking experience, subtle differences may persist into school age, particularly under more demanding conditions such as dual-task walking. However, currently available studies on preterm gait are quite heterogeneous in terms of GA, BW, and/or individual walking experience of included individuals and gait analysis methods employed. Furthermore, evidence regarding gait characteristics in preschool and school-aged preterm children is still relatively scarce, thus hampering a deeper understanding of preterm-born children’s gait development after the toddler stage.

*Conclusion*: Instrumented gait analysis systems and wearable technologies, including inertial measurement units, enable objective and ecologically valid assessment of gait performance. Quantitative gait parameters have shown associations with standardised gross motor scores and may provide additional information beyond milestone history alone. Incorporating structured quantitative gait assessment into longitudinal follow-up programmes could support more detailed monitoring of motor development and inform individualised rehabilitation planning in children born preterm.

**Table:** 

**What is known:** • *Children born preterm generally achieve independent walking later than term-born peers and may show immature gait features, particularly during the first years of life.* • *Most available evidence is heterogeneous and focuses mainly on walking onset or early gait, with limited data extending into preschool and school age.*	
**What is new:** • *This review integrates evidence on gait development from the fi rst independent steps to school age, identifying persistent abnormalities mainly in children born at lower gestational ages, with lower birth weight, or under demanding walking conditions.* • *It highlights the potential role of quantitative gait analysis and wearable inertial sensors as complementary tools for longitudinal neurodevelopmental follow-up and individualized rehabilitation planning.*

**What is known:**

• *Children born preterm generally achieve independent walking later than term-born peers and may show immature gait features, particularly during the first years of life.*

• *Most available evidence is heterogeneous and focuses mainly on walking onset or early gait, with limited data extending into preschool and school age.*

**What is new:**

• *This review integrates evidence on gait development from the fi rst independent steps to school age, identifying persistent abnormalities mainly in children born at lower gestational ages, with lower birth weight, or under demanding walking conditions.*

• *It highlights the potential role of quantitative gait analysis and wearable inertial sensors as complementary tools for longitudinal neurodevelopmental follow-up and individualized rehabilitation planning.*

## Introduction

Human walking is a particular form of locomotion characterised by specific movement patterns of the lower limbs, aiming at translating the body forward, providing propulsion and support, and allowing mobility in the environment according to humans’ needs and curiosity [1, 2]. The toddler stage, between 13 and 36 months, represents the most critical time window for the development of independent walking, being characterised by rapid anatomical growth and neurosensory changes functional to the acquisition of stable bipedal gait [2]. Being able to walk is essential to achieve the true essence of locomotion, which is functional mobility, i.e. the ability to change location according to will and purpose. Providing increased access to the world, independent walking also has a strong impact on the learning process of several everyday skills, such as object and social interactions, social looking, active and passive language, and thus on neurodevelopment as a whole [1].

It is well known that prematurity strongly impacts infants’ neurodevelopment, with extremely preterm-born children experiencing the highest risk of significant impairment in all developmental domains, including cognitive [3] and motor outcomes [4, 5]. Most studies so far have focused on the assessment of early motor development in children born preterm, combining brain magnetic resonance imaging (MRI), general movements (GMs) assessment and the Hammersmith Neurological Examination to predict later motor outcome and the risk of cerebral palsy [6], while a structured body of literature dedicated to the assessment of subsequent motor development, comprising age of walking onset and gait features, is still lacking. Despite being fewer and heterogeneous, studies evaluating later motor achievements generally agree in highlighting that walking onset and gait characteristics differ between preterm and term-born healthy children, potentially limiting the possibility of preterm-born children to fully exploit the developmental benefits of early walking [7]. Further insight into gait assessment and monitoring in preterm-born individuals would aid in defining longitudinal motor developmental trajectories for this vulnerable population and providing targeted rehabilitation therapy to the individuals who are at high risk of deviation from expected standard developmental pathways.

Thus, the primary aim of this narrative review is to describe the development of independent walking in children born preterm, with a focus on age of walking onset and both qualitative and quantitative characteristics of gait in this population, highlighting differences and similarities with typically developing children born at term. The secondary aim is to focus on structured gait assessment and its potential clinical implications during neurodevelopmental follow-up of children born preterm.

## Development of gait in childhood

The following section provides a short introduction on typical gait development, followed by a summary of current evidence on gait development and characteristics in children born preterm.

### Development of gait in healthy children born at term

Human locomotor behaviour starts as early as the 14th week of gestational age (GA), with the first foetal leg movements. After birth, neonates show various types of stepping movements, that are generated by a neural substrate that is very similar to that responsible for GMs and persist up to 2–3 months of age [8]. From 4 to 5 months onwards, infants start to explore the environment using pre-walking solutions, such as rolling movements, prone progression and crawling on hands and knees. After achieving the standing posture, at around 9–13 months for typically developing children, first attempts at walking steps are explored, at first holding an adult’s hand, pushing objects like a “walker” or cruising around the edge of furniture, then, usually around 10–14 months, without any external support [1, 9].

Independent walking lacks a unique definition in literature: most studies use 5 unsupported consecutive steps as a reference, but others consider a variable number from 3 to 10 unsupported steps or the ability to walk without support over a 5-m distance [7]. The lack of clear-cut criteria to define independent walking complicates accurate comparison between different studies. Furthermore, age of walking onset is often based on parental report during routine clinical appointments, which can cause significant measurement bias as parents may interpret “first steps”, “a few unsupported steps” and “independent walking” differently.

Children’s walking, especially in the beginning, is a less mature and less experienced form of movement than that of adults, despite similarities in sensory control. Early stages of independent walking indicate an initial adaptation to poor balance control and limited lower limb control and strength: novice walkers usually make flat-footed contact instead of heel strikes, due to immature foot anatomy and lack of sufficient dorsiflexion during the swing phase. Developing balance and leg strength during single-leg support result in longer double-support phases, shorter single-leg stance times, reduced step length, and slower overall walking speed. To enhance their balance, infants adopt a bent posture with knee flexion, lowering their centre of gravity. They also widen their base of support (BOS) by taking larger steps and externally rotating their feet. Hip movements are often uncoordinated, and arms are held in a “high guard” position instead of swinging in counterphase like in older children or adults, sacrificing energy efficiency for better balance. The challenge of achieving optimal balance doesn’t fully explain the observed dynamic gait features, because similar patterns appear even when infants walk with support before they reach autonomy, thus suggesting a constitutional immaturity of both supported and unsupported gait at its onset [1, 10, 11]. In this context, McCollum et al. described three extreme walking forms that can be observed in the first weeks of independent locomotion: “stepper” infants take small, stable steps, lifting one foot and then quickly placing it back on the ground in a more advanced position, maintaining the trunk vertical and as stable as possible; “twister” infants progress by twisting the trunk around its longitudinal axis with one leg acting as a pivot while the other contributes to trunk angular momentum by rotating forward; “faller” infants project the trunk forward and consequently move the foot in the same direction to advance the BOS and prevent falling, exploiting gravity for onward progression [12]. Children can present with different combinations of these walking forms (e.g., stepper-twister, twister-faller, but never stepper-faller) at the very early stage of walking, to soon converge to a walking form that manifests the typical features of pendulation. The latter type of walking will characterize mature gait, as it depends on appropriate neurological maturation and inter-segmental coordination of the body [11, 13]. Analysing the gait patterns of 20 toddlers during their first 6 months of independent walking with inertial sensors, Bisi et al. found that “stepper” and “twister” strategies were more common than the “faller” one in their cohort, while more pendulum-like patterns appeared in all toddlers after one month of independent walking [14].

The process to reach a more mature form of walking is highly dependent on walking experience, which coupled with external influences (such as caregiving practices) acts by widening or restricting the opportunities to practice. This process determines a progressive selection of preferred walking patterns through the activation of specific muscles instead of others, leading to improved walking proficiency over time [9]. The most significant improvements in walking can be registered during the first three months of independent locomotion, but this maturation process extends far beyond toddler age, with a qualitatively mature, adult-like gait pattern which is usually not achieved before 5–7 years of age [1]. As infants gain more walking experience, they start to take longer, narrower, quicker and less variable steps; heel strikes become evident and double support time decreases; neuromuscular coordination improves; arms begin swinging in counterphase to the legs, helping in balancing trunk rotation and promoting forward movement. In parallel with gait maturation, several anatomical changes support more efficient walking: the spinal curve adapts to improve force distribution, the lower limbs realign to decrease lateral sway, foot arches develop to enhance load acceptance and propulsion [1].

### Development and characteristics of gait in children born preterm

When neuromotor maturation is disrupted or altered, as can occur in children born preterm, walking acquisition and subsequent development may follow different trajectories.

Prematurity is a well-known cause of impaired motor development, ranging from cerebral palsy, the most severe motor disability of childhood, to milder forms, such as developmental coordination disorders (DCD) and delays in the achievement of motor milestones [15, 16]. Independent walking represents one of the most crucial achievements in human motor development, as it is functional to engaging with the surrounding environment, thus promoting visual, cognitive and social skills maturation as well [1]. For this reason, walking attainment is even more critical for children born preterm, given their intrinsic higher risk of neurodevelopmental impairment compared to term-born children. Low GA and birth weight (BW) can significantly impact both the age of walking onset and gait characteristics, outlining a developmental gap between preterm and term-born children [7, 17]. Furthermore, many comorbidities associated with prematurity (such as intrauterine growth restriction, respiratory distress syndrome, bronchopulmonary dysplasia, pneumothorax, necrotizing enterocolitis, sepsis, prolonged oxygen therapy and hospitalization) have been linked to gross motor delay in this population [18].

Information about age of walking onset in preterm-born children is commonly collected during follow-up visits, as it represents one of the most important goals for physiotherapy in these patients. Recent literature reports a delay of approximately one and a half months in walking onset for the preterm population when compared to term-born peers even after adjustment for corrected age (CA), rising to two months delay in studies recruiting children born very preterm and/or with very low BW [7]. A longitudinal study conducted on 60 moderate and late preterm neonates showed a significant correlation between CA of walking onset and GA, birth head circumference (BHC) and total Alberta Infant Motor Scale (AIMS) score at 10 months CA, with a predictive power of 50% for the combined three variables. Specifically, every 1-unit decrease in GA (weeks), AIMS score (points) and BHC (cm) was associated with a delay in the onset of independent walking of 0.296, 0.132 and 0.196 months, respectively [19].

As for preterm gait characteristics, these are not as well defined as in term-born children or in individuals with cerebral palsy. Nonetheless, interest in this research field has grown in recent years, as shown in Table 1, where we report an up-to-date summary of studies on preterm gait characteristics that are currently available to our knowledge. The search has been conducted on Pubmed database during a four-month period ending on 30th November 2025, using “*preterm*”, “*prematurity*”, “*gait*”, “*walking*” and “*spatiotemporal parameters*” as main search terms. All studies conducted on children born preterm (GA < 37 weeks) and providing information about qualitative and/or quantitative features of independent walking in these individuals have been included. Studies focusing exclusively on age of walking onset in preterm-born children with no mention of gait characteristics have been excluded for the specific purposes of this literature search.

**Table 1:** Features and findings of 12 studies on preterm gait characteristics

Author, year, setting (reference)	Study design	Preterm group	Term group	Age at assessment	Assessment tools	Findings
Gorga, 1985, USA [20]	O P M	Preterm AGA infants both with or without medical complications [*n* = 135, HPT 38, SPT 97; mean BW HPT 1573 g, SPT 1261 g]	Healthy full-term AGA infants [*n* = 15]	12 months (CA for the preterm group)	Clinical observation (Neuromotor Behavioural Inventory)	Lower rates of independent walking and less mature walking pattern (less frequent trunk rotation) in PT infants at 12 m CA
Cioni, 1993, Italy [21]	O P M	Preterm AGA infants with no severe postnatal complications [*n* = 25; mean GA 33.8w (SD 2.7); mean BW 2100 g (SD 600)]	Healthy full-term infants [*n* = 25; mean GA 38.4w (SD 1); mean BW 3100 g (SD 300)]	3–4 weeks and 4 months after onset of independent walking (10 successive steps without support)	Videorecording (2 assessors)	Wide early walking pattern variability and frequent gait asymmetries in both FT and PT infants, with no significant differences between the two groups; toe-strike more frequent in PT infants at walking onset and correlated with specific motor features (stepping on tip-toe, inverted kicking pattern and leg extension during suspension)
De Groot, 1997, Netherlands [22]	O CS M	Preterm infants at low risk for later developmental problems [*n* = 33; mean GA 30 + 6w (range 27 + 1–34 + 4); mean BW 1536 g (range 765–2370)]	Healthy full-term AGA infants [*n* = 19]	14 days after onset of independent walking (walking 5 m independently)	Clinical observation and videorecording (Optimal score for walking independently)	Qualitatively different pattern of locomotion in PT infants, with the youngest PT (GA ≤ 32w) and SGA PT overrepresented in the near-poor and poor categories of walking
Jeng, 2008, Taiwan [23]	O P M	Preterm infants without congenital or chromosomal anomalies and without major neonatal diseases that affect neurodevelopment [*n* = 29; mean GA 32w (SD 2.7); mean BW 1800 g (SD 600)]	Healthy full-term AGA infants [*n* = 29; mean GA 38.8w (SD 1.2); mean BW 3300 g (SD 400)]	18 months (CA for the preterm group)	Kinematic analysis	Shorter stride length in PT infants
Kluenter, 2008, Germany [32]	O P M	VLBW preterm infants without CP, unilateral/bilateral blindness and hydrocephalus [*n* = 44; median GA 28.6w (range 23.4–34.1); median BW 1095 g (range 380–1480)	Healthy full-term infants [*n* = 21]	7 years	Balance Master computerized force plate	No differences in step width, length, speed, length asymmetry and end sway during normal walking and tandem walking
Hagmann-Von Arx, 2015, Switzerland [34]	O CS M	VPT infants without significant motor impairment (M-ABC < 16th percentile) [*n* = 44; mean GA 30.1w (SD 2.1); mean BW 1423 g (SD 421)]	Full-term infants without significant motor impairment (M-ABC < 16th percentile) [*n* = 44; mean GA 39.6w (SD 1.5); mean BW 3353 g (SD 429)]	9.5 years	GAITRite during single, dual and triple-task conditions	Systematic decreases in stride velocity variability from VLBW PT infants to PT infants with BW ≥ 1500 g to FT infants, most apparent in dual and triple-task conditions (auditory or verbal fluency task)
Cahill-Rowley, 2016, USA [24]	O CS Multicentric	VLBW preterm infants with GA ≤ 32 weeks [*n* = 81; mean GA 28.8w (SD 2.4]	Healthy full-term infants [*n* = 43; mean GA 39 + 5w (SD 1 + 2)	18–22 months (CA for the preterm group)	GAITRite	Wider step width in PT infants; increased step length asymmetry and step time in PT infants with BSID III motor composite score < 85
Owen-Jones, 2020, France [25]	O CS M	VPT infants without major neurologic disabilities (CP) or cardiac pathology [*n* = 12; mean GA 27.6 w (SD 1.1); mean BW 900 g (SD 300)]	Full-term infants [*n* = 33; mean GA 39.9w (SD 1.5); mean BW 3300 g (SD 600)]	3–6 years	OptoGait during 6MWT	Lower walking speed and normalized stride length in PT infants
Ito, 2021, Japan [33]	O CS M	LPT infants without musculoskeletal, neurologic, auditory, ophthalmologic and cardiorespiratory abnormalities and normal Raven’s Coloured Progressive Matrices and Picture Vocabulary Test [*n* = 22; median GA 36 w (range 34–26); median BW 2360 g (range 1900–2840)]	Healthy full-term infants [*n* = 255; median GA 39w (range 37–41); median BW 3050 g (range 1750–4160)]	6–10 years	Three-dimensional gait analysis system with retro-reflective markers	No differences in gait performance tests (gait speed, step length/height, coefficient of variation, gait deviation index) between FT and PT infants
Albesher, 2022, Australia [7]	O P M	VPT infants without congenital anomalies affecting development [*n* = 112; mean GA 27.8 w (SD 1.4); mean BW 1035 g (SD 252)]	Healthy full-term infants [*n* = 112; mean GA 39.9w (SD 1.19); mean BW 3506 g (SD 450)]	4.5 years (CA for the preterm group)	GAITRite during preferred speed, cognitive and motor dual-task and tandem walking	Preferred speed: higher BOS variability in PT infants; Motor dual-task: higher speed of walking, longer step length and wider BOS in PT infants; Cognitive dual-task and tandem walking: wider BOS in PT infants
Bisi, 2022, Italy [27]	O P M	Preterm infants without CP, congenital malformations and blindness [*n* = 29; median GA 30.5w (range 24–35)]	Healthy full-term infants with GA ≥ 38w [*n* = 17]	2 years (CA for the preterm group)	Wearable inertial sensors (IMUs)	Longer stance and double support time, higher variability of temporal parameters and lower multiscale entropy in PT infants
Topal, 2023, Turkey [26]	O P M	Preterm infants without CP, genetic or metabolic disorders [*n* = 74; mean GA 31.6 w (SD 3.1); mean BW 1764.5 g (SD 589.1)]	Healthy full-term infants [*n* = 38; mean GA 38.18 w (SD 1); mean BW 3111.84 g (SD 395.7)]	3–4 years	GAITRite	EP-VP: shorter step length; MLP: greater step length variability
Albesher, 2025, Australia [29]	O P M	VPT infants born to English speaking parents without congenital abnormalities, CP or intellectual disability (full-scale IQ composite score < 80 on WPPSI-IV) [*n* = 102, of whom 26 at risk for DCD, 76 not at risk for DCD; mean GA at-risk 27.74 w (SD 1.57), no-risk 27.87 w (SD 1.33); mean BW at-risk 1044.12 g (SD 264.93), no-risk 1045.55 g (SD 243.45)]	No term group for comparison	4–5 years (CA for the preterm group)	GAITRite during preferred speed, cognitive and motor dual-task and tandem walking	Preterm newborns at risk for DCD: greater variability in BOS during preferred speed; greater variability in step length and BOS during cognitive dual-task; wider BOS, shorter single support time and higher variability in step time, step length and single support time during motor dual-task; higher cadence, shorter step time and wider BOS during tandem walking
Stagni, 2025, Italy [31]	O P M	Preterm infants [*n* = 55, GA and BW not available]	Full-term infants [*n* = 17, GA and BW not available]	18 and 24 months (CA for the preterm group)	IMUs	Preterm group: longer double support and its short- and long-term variability, reduced entropy and increased recurrence in ML at both 18 and 24 months; increased harmonic ratio in AP and ML components at 24 months Preterm compared to full-term infants with same walking experience: reduced normalised stride and increased double support after 3–5 months of independent walking. Increased short- and long-term variability of double support, increased harmonic ratio, reduced entropy and increased recurrence in ML in the preterm group persist regardless of walking experience. Same alterations but more marked in high risk preterm (GA < 28 w, BW < 1 kg, surgical NEC, IVH grade 3, PVL, BPD of any degree at 36w CA) compared to low-risk preterm infants
Ito, 2025, Japan [35]	O CS M	EP infants without CP, intellectual disability (IQ < 70 on WISC-IV Japanese version), brain MRI anomalies, cardiorespiratory, visual, auditory or musculoskeletal abnormalities [*n* = 10; median GA 27 w (range 22–27); mean BW 857 g (SD 349)]	Typically developing term-born children [*n* = 30; median GA 39w (range 37–41); mean BW 3028 g (SD 355)]	6 years	Three-dimensional gait analysis system with retro-reflective markers	No significant differences in spatiotemporal gait parameters between the two groups, but lower GDI and higher GDI symmetry ratio (indicating more pronounced GDI asymmetry) in the EP group

*AGA*, Appropriate for gestational age; *AP*, Antero-posterior; *BOS*, Base of support; *BPD*, Bronchopulmonary dysplasia; *BW*, Birth weight; *CA*, Corrected age; *CP*, Cerebral palsy; *CS*, Cross-sectional; *DCD*, Developmental coordination disorder; *EP*, Extremely preterm (GA < 28 weeks); *FT*, Full-term; *GDI*, Gait Deviation Index; *HPT*, healthy preterm; *IVH*, Intraventricular haemorrhage; *LPT*, Late preterm (GA 34-36w); *M*, Monocentric; *M-ABC*, Movement Assessment Battery for Children; *ML*, Medio-lateral; *MLP*, Moderate and late preterm (GA 32–34 weeks); *NEC*, Necrotizing enterocolitis; *O*, Observational; *P*, Prospective; *SPT*, Sick preterm; *VLBW*, Very low birth weight (BW < 1500 g); *VPT*, Very preterm (GA < 32 weeks); *WISC-IV*, Wechsler Intelligence Scale for Children, 4th edition; *WPPSI-IV*, Wechsler Preschool and Primary Scale of Intelligence, 4th edition; *6MWT*, 6-min walking test

The first attempts to characterize preterm gait have adopted qualitative assessments such as laboratory observation and videorecording, reporting a less mature walking pattern during the first months after the onset of independent walking. This is characterised by less frequent trunk rotation and more frequent toe-strike, the latter probably being caused by underdeveloped balance due to immature foot dorsiflexion [20–22]. De Groot et al. suggested a clinical scoring system for independent walking to classify children into 4 categories of walking proficiency (optimal, near optimal, near poor and poor) according to 15 items describing their gait pattern. After applying this score to a cohort of 33 preterm-born children, the authors described a higher prevalence of “near poor” and “poor” pattern of walking in the youngest preterms (GA ≤ 32 weeks) and in those who were small for GA, probably because of a higher difficulty in regulating muscle power, especially during flexion–extension movements, compared to children with a higher GA and/or BW [22].

Subsequent research has focused on quantitative characterization of preterm gait, mainly using instrumented walkways (such as the GAITRite system) to define spatiotemporal parameters in these individuals. Quantitative studies may be subcategorised according to the different timing of evaluations: first months after onset of independent walking, preschool, and school age.

During the first months of independent walking, children born preterm show shorter stride length and wider step width than full-term peers, which may represent a strategy to compensate for decreased balance and immature postural control in children born with a lower GA [23, 24]. Cahill-Rowley et al. analysed gait differences at 18–22 months CA between very low BW (VLBW, BW < 1500 g) preterm-born children, subcategorised according to a high (**≥ **85) or low (< 85) Bayley Scales of Infant and Toddler Development III motor composite score, and full-term peers: lower-scoring preterm-born children demonstrated significantly increased step width and step length asymmetry compared to term-born typically developing toddlers, and increased step time in respect to higher-scoring preterm and full-term peers. All other gait parameters showed similar means between full-term and preterm participants, reflecting an overall good motor outcome for most preterm-born children, but with wider variations in single gait parameters for the preterm population, probably derived from individual patients experiencing some degree of motor delay [24].

Preschool evaluations have reported the persistence of some of the differences mentioned above in gait parameters between preterm and term-born children, such as shorter stride and step length [25, 26]. Bisi et al. assessed preterm gait at 2 years CA using wearable inertial sensors, describing longer stance and double support time, higher variability of temporal parameters, and lower multiscale entropy in the preterm group compared to term-born controls: these findings point to a simpler and less mature form of gait in children born preterm, with the need to find a stabilizing energy through gait adjustments to compensate for the delayed maturation of trunk control [27]. One study analysing the walking performance of children born very preterm aged 3–6 years during the six-minute walk test (6MWT) with OptoGait (an infrared-based technology) reported a decreased walking performance for preterms, with lower walking speed and an overall lower walking distance associated with increased muscular oxygen extraction measured with near-infrared spectroscopy, highlighting preterm-born children’s reduced functional capacity due to muscular maladjustment during motor tasks [25]. Albesher et al. used the GAITRite system to assess gait characteristics of children born preterm with GA < 30 weeks during preferred-speed walking, cognitive and motor dual-task and tandem walking at 4.5 years CA. As hypothesised by the authors, performing a concomitant task or walking in tandem determined the most significant differences in spatiotemporal gait parameters between the preterm group and the term controls: during cognitive dual-tasks, preterm toddlers gave fewer verbal accurate responses and walked with a wider BOS; during motor dual-tasks, they walked faster, with longer step lengths and a wider BOS, even though the number of dropped balls was similar between preterm and term individuals; during tandem walking, the preterm group exhibited a wider BOS and higher BOS variability compared to term-born peers. These findings underline that preterm toddlers at 4.5 years CA still need to resort to coping strategies to avoid a loss of balance during walking, especially under dual-task conditions [28]. A secondary analysis on a subgroup of participants from the same original cohort identified a vulnerable subcategory of very preterm children at higher risk of gait impairment, represented by children at risk for DCD (defined as having a Movement Assessment Battery for Children 2nd edition score ≤ 16th percentile, full-scale composite intelligence quotient ≥ 80 on the Wechsler Preschool and Primary Scale of Intelligence 4th edition and no diagnosis of CP). While these individuals, examined at 4–5 years CA, showed comparable gait patterns to children born very preterm and not at risk for DCD during preferred speed walking, dual-task and tandem conditions revealed a significant gap between the two groups of children, with wider BOS and shorter single support time in motor dual-tasking and wider BOS and higher cadence during tandem walking in children at risk for DCD. In light of these findings, the authors point out that early detection of this subset of more vulnerable patients is desirable both to inform targeted referral to rehabilitation programmes and to adapt daily routine and sport activities to minimise the impact of specific dual-task conditions on gait performance, preventing unnecessary loss of balance and fall-related injuries [29].

From these data, it seems that preterm-born preschoolers still exhibit some degree of immaturity in their gait pattern, especially in case of more vulnerable subsets of patients and during highly-demanding tasks, but the different timing of independent walking onset, and thus a different walking experience at the time of assessment, between preterm and term-born children should be taken into consideration, as it could account for at least some of the observed differences between these two populations, in addition to the constitutional gap related to differences in GA and BW. Even though the mentioned studies lack patient stratification by walking experience, Jeng et al. reported an improvement in some gait parameters in the preterm population with increasing walking experience (i.e. increased walking speed, stride length and percentage of swing time and decreased percentage of stance time, asymmetry ratio of stance and swing time) [23]. Conversely, Cahill-Rowley et al. pointed out that the impact of walking experience on gait parameters is significant only during the first 2 months after the onset of independent walking, with only a weak correlation with step length asymmetry in preterm toddlers [30]. A recent study from Stagni et al. compared gait metrics recorded with inertial measurement units (IMUs) in a cohort of preterm-born children and term-born peers with the same walking experience, highlighting the persistence of many alterations in spatiotemporal and non-linear gait metrics in the preterm group, though participants were assessed only as early as at 18 and 24 months of CA [31].

Significant improvements in gait features have been reported in preterm-born children assessed at school age, with none or only slight differences when compared to term-born peers. Specifically, a cohort of 44 VLBW preterm-born children assessed with the Balance Master force plate at 7 years of chronological age showed comparable step width, step length, step speed, step length asymmetry, and end sway during normal walking and tandem walking to the term-born control group [32]. Similarly, Ito et al. did not observe any significant difference in gait speed, normalised step length, coefficient of variation, and gait deviation index (GDI) between a group of 22 late preterms and term-born healthy children assessed at 6–10 years of chronological age with retro-reflective markers while walking at usual speed [33]. Only one study on 44 children born very preterm (of whom 22 VLBW) assessed at 9.5 years of chronological age with the GAITRite system reported a systematic increase in stride velocity variability from term-born children to preterms with BW ≥ 1500 g to VLBW preterm patients, which was most apparent in dual- and triple-task conditions (auditory or verbal fluency tasks). The latter finding highlights a poorer walking performance even at older ages for children born preterm with a lower BW [34]. Furthermore, a recent study on a small cohort of children born extremely preterm (GA < 28 weeks) assessed at 6 years with three-dimensional gait analysis system showed that, even though there were no significant differences in spatiotemporal gait parameters compared to age-matched typically developing term-born children, the preterm group exhibited lower GDI scores and more pronounced GDI asymmetry. This finding indicates reduced quality of gait in this population and brings back the attention on gait quality assessment, which integrated to quantitative gait parameters might favour a more accurate and comprehensive understanding of motor performance in these patients. To note, decreased gait quality was significantly associated with lower BW and subsequent weight gain at 36w of post-menstrual age, suggesting that nutritional management during the neonatal period might influence motor outcomes in school-aged children [35].

In conclusion, available literature on preterm gait characteristics describes an immature walking pattern for most preterm-born children especially during the first months of independent walking and during preschool years, with the possibility to catch-up with increasing walking experience and increasing age, particularly for children born preterm with a higher BW (**≥ **1500 g) and/or GA (> 34 weeks). However, care should be taken to differentiate these low-risk patients from specific subgroups of high-risk preterm-born children, such as those with extremely low BW and/or GA or with additional risk factors for neuromotor impairment (such as DCD traits or postnatal growth faltering). Indeed, studies focusing specifically on these more vulnerable categories are still scarce and the available literature underlines the persistency of more immature features of gait through preschool and school age in these patients. Further research on wider cohorts, including higher-risk individuals, and with standardised protocols is needed to expand our current understanding of motor development trajectories in children born preterm.

## Gait assessment in children born preterm

### Gait characteristics and assessment

From the first baby steps until mature gait is achieved, walking features can be described using measurable parameters. Integrating quantitative gait assessment in the overall evaluation of toddlers and children’s well-being can add valuable information from a global health perspective, given the established relationship between motor skills and not only physical performance, but also social participation, academic achievement, cognitive skills and mental health [36–38].

To interpret the quantifiable differences in walking onset and gait characteristics between typically developing children and those at risk for motor and neurodevelopmental impairment, it is first necessary to briefly introduce the methods commonly described in the literature for quantitative gait assessment [2, 7, 27, 39, 40].

Each gait cycle, referred to as a stride, goes from the contact of one foot with the ground to the following contact of the same foot with the ground and includes two phases: the stance phase, from the contact of the foot with the ground to its following lift off, and the swing phase, from the lift of the foot from the ground to the following contact with the ground. Stride time can also be divided into single support (time spent with only one foot in contact with the ground) and double support duration (time spent with both feet on the ground). The time interval between the initial contact of one foot and the subsequent initial contact of the opposite foot is a step.

Over each stride, several spatial and temporal characteristics can be observed and quantified by means of measurable descriptive parameters of gait performance, many of which are consistently reported in the scientific literature [2, 7, 27, 39, 40], such as spatiotemporal parameters (e.g., stride and step length and duration, gait velocity, stance, swing and double-support duration relative to stride time, cadence), kinematic (e.g., joint angles), and dynamic parameters (e.g., ground contact force components, joint moments and powers) [41]. These parameters, and how they change with maturation, play a fundamental role in understanding motor development [42].

Beyond these traditional motion analysis descriptors, in recent years a number of linear (e.g., variability indexes, harmonic ratio) and non-linear metrics (e.g., Lyapunov exponents, complexity, recurrence analysis), calculated on temporal parameters and kinematic variables, have been proposed for the characterization of specific aspects of motor performance and control (e.g., variability, fluidity, regularity, stability, complexity, automaticity) [43, 44]. These novel metrics complement traditional gait parameters by offering a more complete picture of how stable, robust, adaptable, and mature a subject’s walking pattern is, providing particularly valuable information when studying developmental changes or detecting early signs of motor difficulties.

Over the years, various techniques have been employed to enable the analysis of measurable gait parameters [37]. Laboratory assessment using 3D digital motion capture of kinematics and kinetics allowed collection of detailed biomechanical information, even though these types of analyses remain difficult to integrate in standard clinical assessments and of limited feasibility for non-cooperative subjects. In recent years, portable devices allowed for easy ambulatory assessment, supporting community-based and ecologically valid evaluation of motor performance, with direct clinical implications. These portable devices can be broadly divided into two main categories: i) environmental (e.g., markerless video analysis; sensorised mats and platforms); ii) wearables (e.g., inertial measurement units, pressure insoles and sensors) [1]. The first group comprises video-based systems (similar to KinectTM), which measure kinematic and spatiotemporal parameters from traditional video recordings, and pressure sensor mats (such as GAITRite), force platforms, and optoelectronic bars, that allow measurement of spatiotemporal parameters [45–47]. The second group does not require any sensorisation of the environment and includes low-tech strategies, such as inked shoe tabs, and high-tech measuring devices, such as inertial sensors [1, 47, 48]. These technology-based approaches enable the quantitative description of gait, allowing the characterisation of typical gait development, the identification of atypical trajectories and the longitudinal monitoring of walking performance, which might be particularly useful in children at high risk of motor developmental impairment, such as children born preterm with extremely low GA and/or BW or comorbidities affecting neuromotor development [14, 37, 41].

In particular, inertial measurement units (IMUs) are a very promising, easy-to-use, lightweight, and non-intrusive technology that employs accelerometers and gyroscopes to measure linear acceleration and angular velocity of the walking patient [38, 49]. Nowadays, despite being widely used as a reliable method for real-time gait analysis in adults and adolescents with gait impairments (amputees, people with neurological diseases or traumatic brain or spinal cord lesions), their application in the paediatric field is limited and mainly related to research activities [38, 50]. Nonetheless, IMUs represent an attractive alternative to traditional laboratory gait analysis, especially for the assessment of children, because they are easy to wear, can be hidden under clothes and do not restrict the natural movement pattern of the child. Recent research reported a first database of normative values for spatiotemporal parameters derived by inertial sensors in 162 typically developing children and young adults 5–30 years old, offering a reference database to compare data from patients with gait impairments and their responses to interventions [51]. However, paediatric normative data for smaller children and validated on larger cohorts of individuals are still lacking and clinical cut-off values for accurate interpretation of spatiotemporal gait parameters have not been clearly defined yet. Moreover, technical issues such as internal algorithm variability should be taken into account when considering IMUs reliability, highlighting the need for further technological refinement and standardization of these tools for paediatric use.

### Integration of gait assessment into neurodevelopmental follow up of children born preterm

The findings presented above raise relevant questions about whether, how, and for how long gait development should be monitored in children born preterm, and how walking characteristics can inform clinical follow-up.

After hospital discharge, high-risk preterm-born children are usually enrolled into neurodevelopmental follow-up programmes held by multidisciplinary teams that monitor clinical, motor, cognitive, language, and social-behavioural development throughout the first years of life, aiming at the earliest possible recognition of any delay in specific developmental domains and at the prompt initiation of tailored neurodevelopmental interventions [52]. Gross motor skills are one of the first developmental domains to emerge, and early motor assessment during the first 6 months of CA is crucial for prompt identification of high risk of motor impairment (either with or without cerebral palsy) [53–55]. Motor development evaluations usually comprise GMs assessment, the collection of a thorough motor milestones history, and standardised assessments of neuromotor function with validated scoring systems [54–58].

Currently, structured gait assessment is not routinely included in the neuromotor follow-up of children born preterm. Nevertheless, we believe that a tailored evaluation of walking patterns in this high-risk population could be very useful to inform clinical decisions on physiotherapy and rehabilitation strategies, promoting a holistic approach to children’s motor competences and contributing to clinicians’ ability to refer patients to appropriate (re-)habilitation interventional programmes.

Wearable technologies, particularly IMUs, appear especially promising in this context. As already discussed, the studies by Bisi et al. demonstrated how these non-invasive sensors can capture meaningful features of motor behaviour in preterm infants and children, highlighting their potential to enrich current assessment practices and support clinical monitoring [27, 49]. Similarly, Cahill-Rowley et al. sustained the possibility of introducing wearable sensors for motor evaluations of children born preterm in the clinic setting [59].

Recent research has focused on the relevance of GMs assessment, especially through longitudinal evaluations, to describe individual trajectories over time, as an early predictive marker of impaired motor and cognitive outcome in preterm-born children [56, 60]. In this perspective, quantitative assessment of gait patterns in this population could represent a possible “late” marker of gross motor delay, increasing the accuracy for the prediction of overall motor developmental impairment and leading the way for increasingly detailed motor follow-up of these patients. To our knowledge, only one study analysed the relationship between BSID III motor scores and spatiotemporal gait parameters (recorded with the GAITRite system) in a cohort of 81 VLBW preterm-born children assessed at 18–22 months of CA. The authors of the study demonstrated positive correlations between motor composite and gross motor scores and velocity, and right side step length, and negative correlations with cycle time, right side stance, and bilateral step time. Additionally, gross motor score significantly correlated with reduced step length asymmetry and increased left side step length [24]. These findings suggest that the evaluation of spatiotemporal gait parameters could be useful to evaluate gross motor development after the onset of independent walking, integrating currently existing standard gross motor assessment scores. With this aim, Cahill-Rowley et al. developed the Toddle Temporal-spatial Deviation Index (Toddle TDI), a single objective score representing toddler gait performance defined as the deviation in spatiotemporal gait parameters (recorded with a GAITRite pressure-sensitive mat) from the average values of a cohort of 42 typically developing children aged 18–22 months. The application of the Toddle TDI to 81 VLBW preterm-born children (GA ≤ 32 weeks) showed lower mean TDI values in the preterm population compared to typically developing toddlers, with significantly lower values in preterms with a BSID III composite motor score < 85 compared to those scoring ≥ 85 and typically developing toddlers. Furthermore, the Toddle TDI demonstrated a stronger correlation with gross motor and composite motor BSID III scores than any single gait parameter, while no relationship was found with fine motor scores, underlining both the sensitivity and specificity of this composite score as a gross motor measure. The authors plan future application of the Toddle TDI on larger cohorts of both term and preterm-born children to further test its validity and reliability and suggest usage of other tools for quantitative gait assessments, such as IMUs, to exploit TDI applications in the clinical setting.

If the association between quantitative assessment of gait parameters and motor development in children born preterm is confirmed by future research, we believe it could then be possible to aim for a longitudinal description of motor developmental trajectories which would include clinical and neurological data, GMs characteristics, and gait features [61]. Longitudinal multimodal integration of clinical and instrumental data is desirable as it should be more accurate in predicting motor and/or global neurodevelopmental outcomes in this population. This integrated approach would be most beneficial for preterm-born children at the highest risk of motor impairment, either due to constitutional risk factors (extremely low BW and/or GA) or comorbidities affecting neurodevelopment, as it would potentially increase clinicians’ ability to recognise early signs of motor impairment. Children born preterm with an early diagnosis of motor impairment, if promptly addressed and treated, hold the highest potential for improvement, with a significant impact on global developmental prognosis. In view of their usually complex clinical histories, these high-risk preterms might present with different degrees of delay in more than one developmental domain, which can often cause standard neuromotor developmental assessments to be quite difficult to complete, owing to their long times of administration. Wearable technologies, such as IMUs, could aid in obtaining clinically informative and ecologically valid motor data in a less stressful way, especially in case of less compliant patients, and can be used during routine follow-up appointments, as they do not interfere significantly with clinical workflow.

In addition, from an interventional point of view, studies about gait in children born preterm have highlighted their immature quality of gait at onset, thus underlining the need for continuing clinical monitoring and physiotherapy well beyond the acquisition of independent walking. The ability to walk without support represents a crucial motor milestone for typically developing children, but it should not be considered necessarily in the same way for preterm-born children. These individuals experience potential delays in most developmental areas, and even though the achievement of independent walking should still be regarded as a positive accomplishment, it should not be seen merely as an endpoint, but rather more as a foundation for future motor and developmental achievements, meaning this new motor ability can help them maximise all the benefits from their interventional programmes. Furthermore, quantitative gait parameters, particularly when linked to neuromuscular impairment, may support physical rehabilitation approaches, helping clinicians select the best strategies and offering a clinical benchmark during and after physiotherapy to better define motor outcomes and improvements, both for longitudinal monitoring of the single patient and to inform comparison between different interventional practices [47, 62–64].

## Conclusions

Independent walking represents a fundamental motor milestone in children’s development and, given its positive impact on global neurodevelopment, it is even more crucial in children born preterm. Nonetheless, walking onset is often delayed in this population and preterm gait usually displays some immature features when compared to that of term-born typically developing toddlers, though findings may vary according to different GA and/or BW, with the smallest infants usually presenting with the most significant impairments. For these reasons, quantitative gait assessment might be considered as a useful complementary tool together with routine longitudinal neuromotor evaluations in the context of neurodevelopmental follow-up for preterm-born children, profiting from emerging portable technologies such as IMUs, which allow non-invasive, ecologically valid, quick and reproducible assessments of gait performance in the clinical setting. Future research should aim at conducting longitudinal studies on wider cohorts of patients, possibly including children born with extremely low BW and/or GA, and employing standardised protocols for motor assessment, in order to define normative values for quantitative gait parameters and meaningful clinical cut-offs for data interpretation. Moreover, efforts should be directed at investigating correlations between measurable gait parameters and gross motor assessment scores in the preterm population, which may help identify possible early gait biomarkers of motor impairment and design clinical scoring systems based on quantitative assessment of independent walking, thus contributing to clinicians’ ability to predict neuromotor outcomes in these high-risk patients and to refer them for early neurodevelopmental interventions.

## Data Availability

No datasets were generated or analysed during the current study.
